# Probiotic *Lactobacillus paracasei* A221 improves the functionality and bioavailability of kaempferol-glucoside in kale by its glucosidase activity

**DOI:** 10.1038/s41598-018-27532-9

**Published:** 2018-06-18

**Authors:** Yosuke Shimojo, Yusuke Ozawa, Toshihiko Toda, Kentaro Igami, Takahiko Shimizu

**Affiliations:** 1 R&D Group, Production Development Division, Nagase BeautyCare & Co., Ltd., 2-2-3 Murotani, Nishi-ku, Kobe, Hyogo 651-2241 Japan; 2 Department of Clinical Cell Biology and Medicine, Chiba University Graduate School of Medicine, 1-8-1 Inohana, Chuo-ku, Chiba, 260-8670 Japan; 3 Department of Advanced Aging Medicine, Chiba University Graduate School of Medicine, 1-8-1 Inohana, Chuo-ku, Chiba, 260-8670 Japan

## Abstract

The interplay between food components and gut microbiota has been considered an important factor affecting the functionality of health-promoting foods. In this study, the effects of the probiotic *Lactobacillus paracasei* A221 on the functionality and bioavailability of kaempferol-3-o-sophroside (KP3S), a kaempferol-glucoside contained in kale, were investigated *in vitro* and *in vivo*. Unlike the type strain NBRC15889, the A221 strain converted standard KP3S as well as the kaempferol-glucosides in kale extract into kaempferol (KP). Using an intestinal barrier model, treatment with A221 significantly improved the effects of kale extract on the barrier integrity *in vitro*. KP, but not KP3S, clearly induced similar effects, suggesting that KP contributes to the functional improvement of the kale extract by A221. Pharmacokinetics analyses revealed that the co-administration of A221 and KP3S significantly enhanced the amount of deconjugated KP in murine plasma samples at 3 h post-administration. Finally, the oral administration of KP to *Sod1*-deficinet mice, which is a good mouse model of age-related disease, clearly ameliorated various pathologies, including skin thinning, fatty liver and anemia. These findings suggest that *Lactobacillus paracasei* A221 is effective for enhancing the anti-aging properties of kaempferol-glucosides by modulating their functionality and bioavailability through the direct bioconversion.

## Introduction

Recent studies on the health-promoting functionality of food have shown the importance of the interplay between the gut microbiota and several classes of food components, such as polyphenols or saponins^1–4^. One famous example is the triterpene saponin contained in the traditional Chinese medicinal herb ginseng (*Panax ginseng* Mayer). It has been reported that the bioavailability of ginseng saponins, called ginsenosides, is relatively low, and the gut microbiota are responsible for increasing the bioavailability as well as the pharmacological activities of these components^5,6^.

It is well documented that certain intestinal bacteria are able to convert the ginsenoside Rb1, a major component of ginseng, into the pharmacologically active component M1 (also referred to as Compound K)^7–10^. One of the unique roles of gut microbiota is to hydrolyze the glucose moieties of Rb1 so that the produced metabolite, M1, can be more bioavailable, exerting its pharmacological activity at different sites of the body. There are, however, inter-individual differences in the body’s ability to produce M1^11,12^. Thus, the standardization of such differences is of great importance in order to maximize ginseng’s potential health benefits in all individuals.

*Lactobacillus paracasei* A221, a probiotic strain, was first isolated from a fermented food and found to be able to improve ginseng’s benefits^13,14^. This strain is unique in its Rb1-hydrolyzing ability and can produce M1 without the help of other strains. Beta-glucosidase expressed in the A221 strain is suspected to be responsible for its hydrolyzing activity. Ingesting probiotics with a high hydrolyzing ability for certain classes of saponins might help modulate the functionality of traditional medicines like ginseng. However, despite mounting evidence supporting the involvement of gut microbiota in the efficacy of Chinese medicinal herbs^15^, studies on the interplay between the gut microbiota and the components of more general foods, such as vegetables, are scarce.

Kale, a *Brassica* species, is a common vegetable consumed by Japanese people in their daily lives and is reported to contain several flavonoid glucosides, such as kaempferol-3-o-sophroside (KP3S) and kaempferol-3-o-sophoroside-7-o-glcuoside (KP3S7G)^16–18^. Since these components are glucosylated, there may be hidden functional potentials in these phytochemicals, and biological activities may be induced by the gut microbiota as well as by certain types of unique probiotics.

In this study, the unique property of *Lactobacillus paracasei* A221 in the metabolism of kaempferol-glucoside, namely KP3S, was investigated, and the relationship between the structural change of flavonoid-glucoside and its functionality was assessed using the *in vitro* intestinal barrier model of the Caco-2 cell line. In addition, the effect of A221 on the bioavailability of KP3S was also verified *in vivo* using C57/B6 mice. Finally, the effect of KP, which is expected to be produced through the bioconversion of kaempferol-glucosides, was tested in hairless *Sod1*-deficinet (*Sod1*^−/−^) mice, a good mouse model of aging phenotypes to obtain clues on the significant role of KP in age-associated disorders.

## Results

### Probiotic *Lactobacillus paracasei* A221 converted KP3S into KP

Because numerous flavonoids, such as kaempferol and quercetin, are usually contained in vegetables in the form of glucosides, their health-promoting functionalities may be somewhat masked compared to their respective aglycones. To evaluate this possibility, we investigated the need for the bioconversion of flavonoid glucosides by intestinal bacteria. The probiotic lactic acid bacteria and kale, which has been reported to contain kaempferol-glcuosides such as KP3S and KP3S7G, were chosen as the model bacteria and food material, respectively^17^.

**Figure 1:**
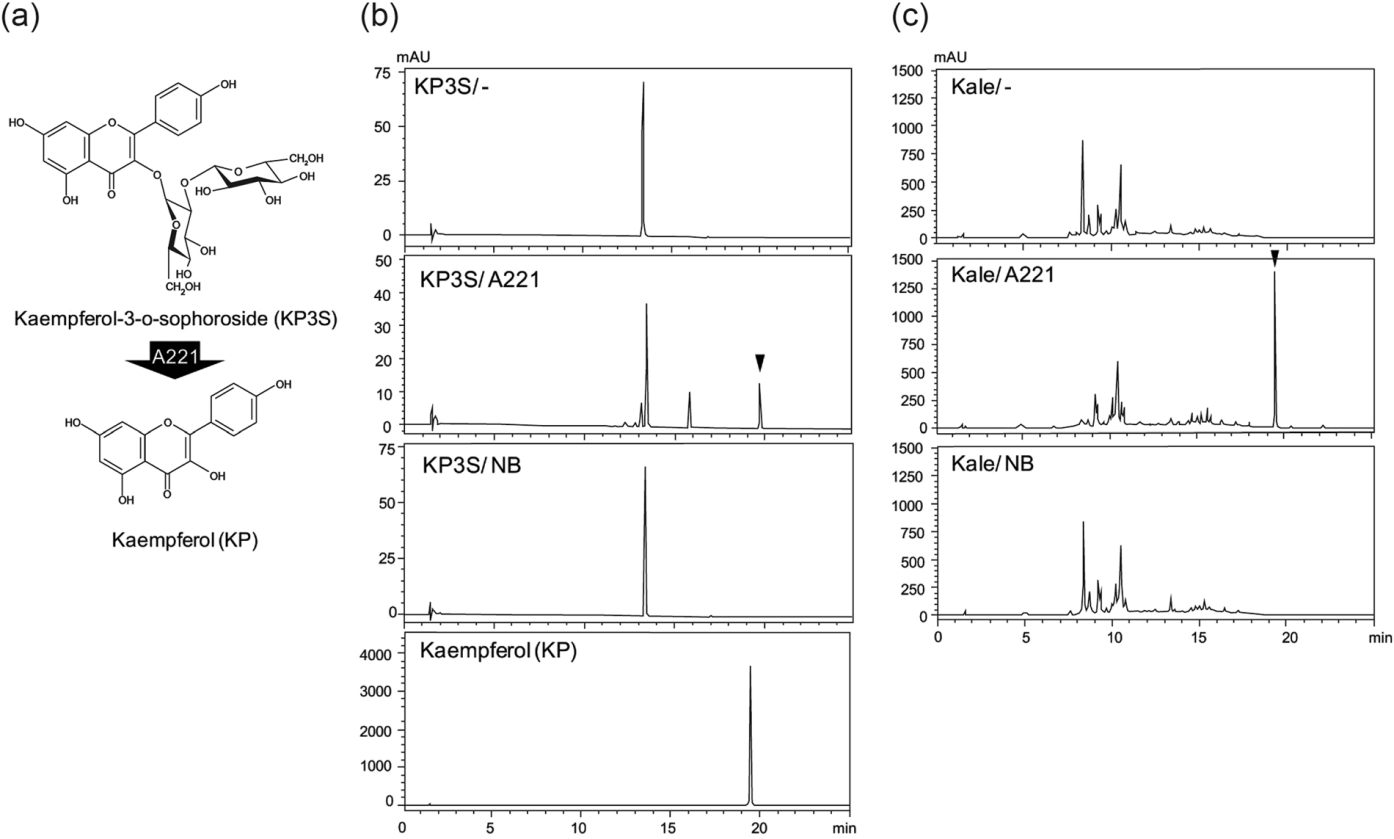
Effect of the *Lactobacillus paracasei* A221 strain on the bioconversion of kaempferol-3-o-sophoroside (KP3S) and kale components. (**a**) Chemical structure of kaempferol-3-o-sophoroside and schematic illustration of the bioconversion into kaempferol (KP) by the A221 strain. (**b**) The A221 strain was incubated with KP3S for 72 h, and the direct effect of A221 on the metabolism of KP3S was assessed with HPLC. (**c**) The A221 strain was incubated in water extract of kale for 72 h and subjected to an HPLC analysis. Arrowhead indicates the peak of KP.

Before evaluating the change in kale’s components, the direct effect of lactic acid bacteria treatment on KP3S was investigated (Fig. 1a). KP3S is composed of an aglycone and a disaccharide part, which is directly attached to position 3 of the C-ring via O-linkage. The disaccharide part consists of two glucose molecules that are linked to each other with beta-1,2 glycoside linkage, resulting in a sophoroside type disaccharide. Co-incubation of this flavonoid glucoside with *Lactobacillus paracasei* A221 for 72 h showed a specific peak at 19.5 min, which corresponded to the peak of the aglycone KP (Fig. 1b). However, treatment of KP3S with *Lactobacillus paracasei* NBRC15889, the type strain of *Lactobacillus paracasei*, showed no such changes, suggesting that the conversion of KP3S into KP is strain-dependent.

**Figure 2:**
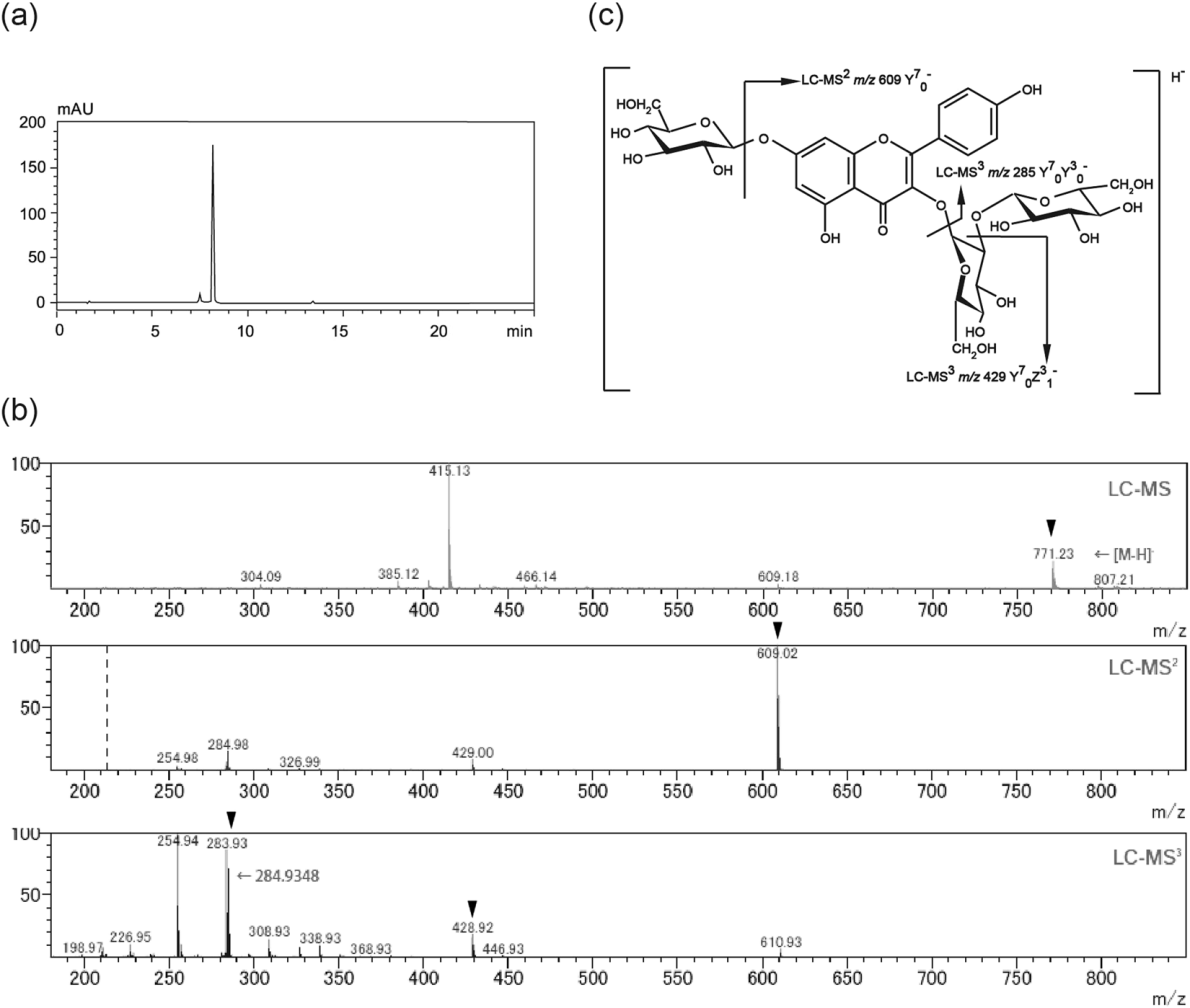
The LC-MS^3^ analysis of the peak at 8.2 min for kale water extract. (**a**) The compound at 8.2 min was purified and subjected to an HPLC analysis. (**b**) Full scan spectra (LC-MS) of the compound at 8.2 min as well as LC-MS^2^ and LC-MS^3^ spectra are shown. Arrowhead indicates unique ions shown by LC-MS, LC-MS_2_ and LC-MS_3_ analyses of KP3S7G. (**c**) The deduced chemical structure and fragment pattern of the compound at 8.2 min.

Because we detected a direct role of the A221 strain in the metabolism of KP3S, we next evaluated changes in the components of kale. As stated in the Methods section, treating kale water extract with A221 for 72 h lead to the disappearance of the main peak at 8.2 min and the appearance of a new peak of KP (Fig. 1c). For the peak at 8.2 min, an LC-MS analysis revealed the chemical formula to be C_33_H_40_O_21_, which corresponds to the formula of KP3S7G. Further results of the LC-MS^3^ analysis showed three fragmented ions of m/z 609, 429 and 285, suggesting that the peak compound at 8.2 min was KP3S7G, the other type of kaempferol-sophoroside, with an additional glucose moiety at position 7 of kaempferol aglycone (Fig. 2). Kale water extract treated with NBRC15889 did not show any apparent changes. These results show that the A221 strain is able to convert KP3S and KP3S7G in kale into aglycone by its unique beta-glucosidase activity.

### A221 regulated the barrier-enhancing properties of kale water extract, including kaempferol-glucosides

**Figure 3:**
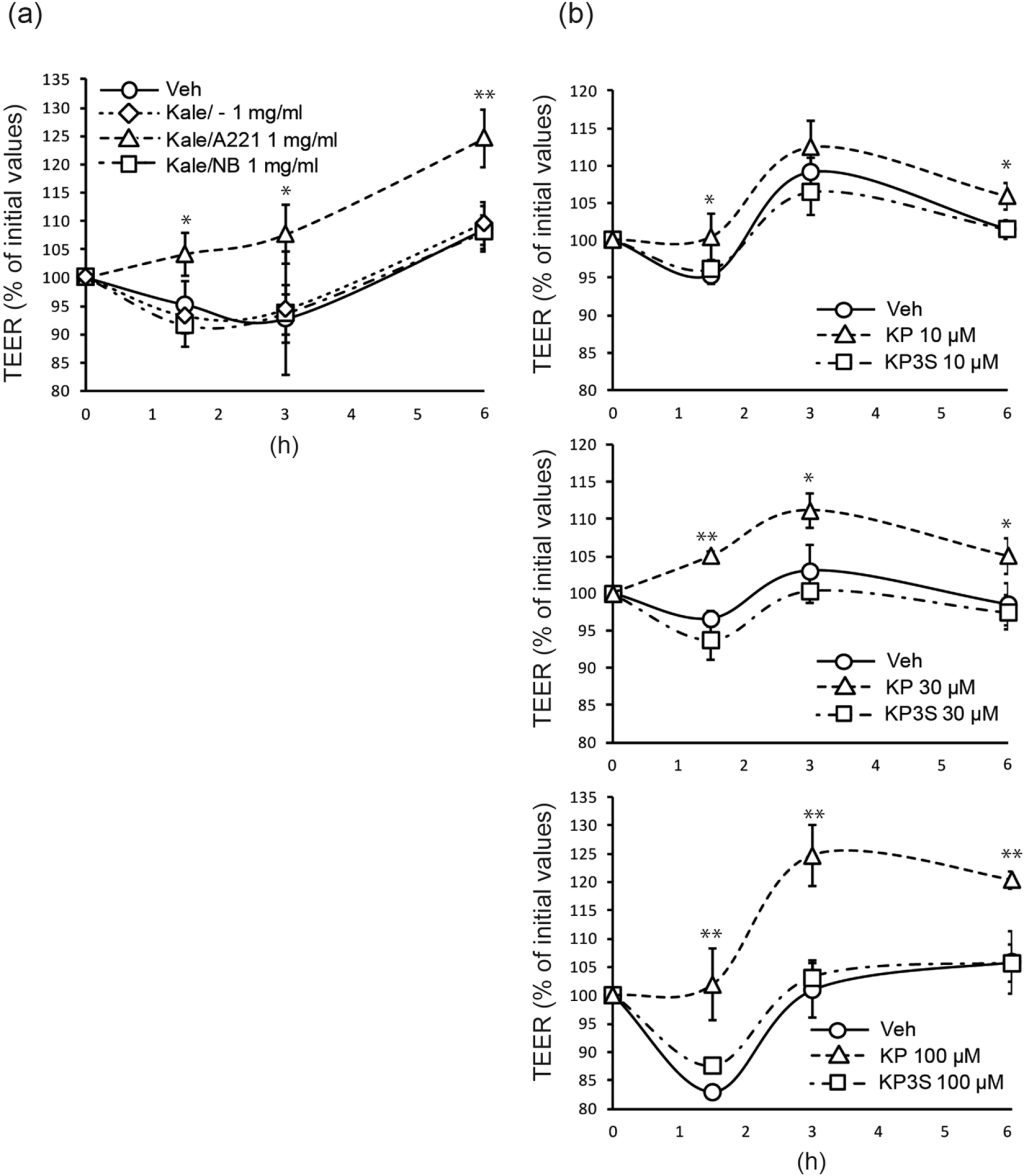
The role of A221 in improving the barrier-enhancing property of kale water extract. (**a**) The intestinal barrier-enhancing property was assessed for each test sample as described in the Methods section using a Caco-2 *in vitro* model. The Caco-2 cell monolayer was differentiated and treated with test samples on the apical side, and TEER values were recorded at the indicated time intervals. (**b**) KP and KP3S were administered to Caco-2 cells, and the TEER values were recorded as in the previous experiment. *P < 0.05 vs. Vehicle. **P < 0.01 vs. Vehicle.

Using a Caco-2 intestinal barrier model, the efficacy of lactic acid bacteria-treated kale extracts was investigated in a functional analysis. As shown in Fig. 3a, treating Caco-2 cells with NBRC15889-treated kale extract or the kale extract without lactic acid bacteria treatment did not show any clear changes in the transepithelial electrical resistance (TEER) values compared to the vehicle control group at any time intervals. A221-treated kale extract, however, significantly increased the TEER values at 1.5, 3 and 6 h post-treatment, suggesting that the efficacy of kale extract on the barrier integrity was enhanced by the treatment with this strain.

**Figure 4:**
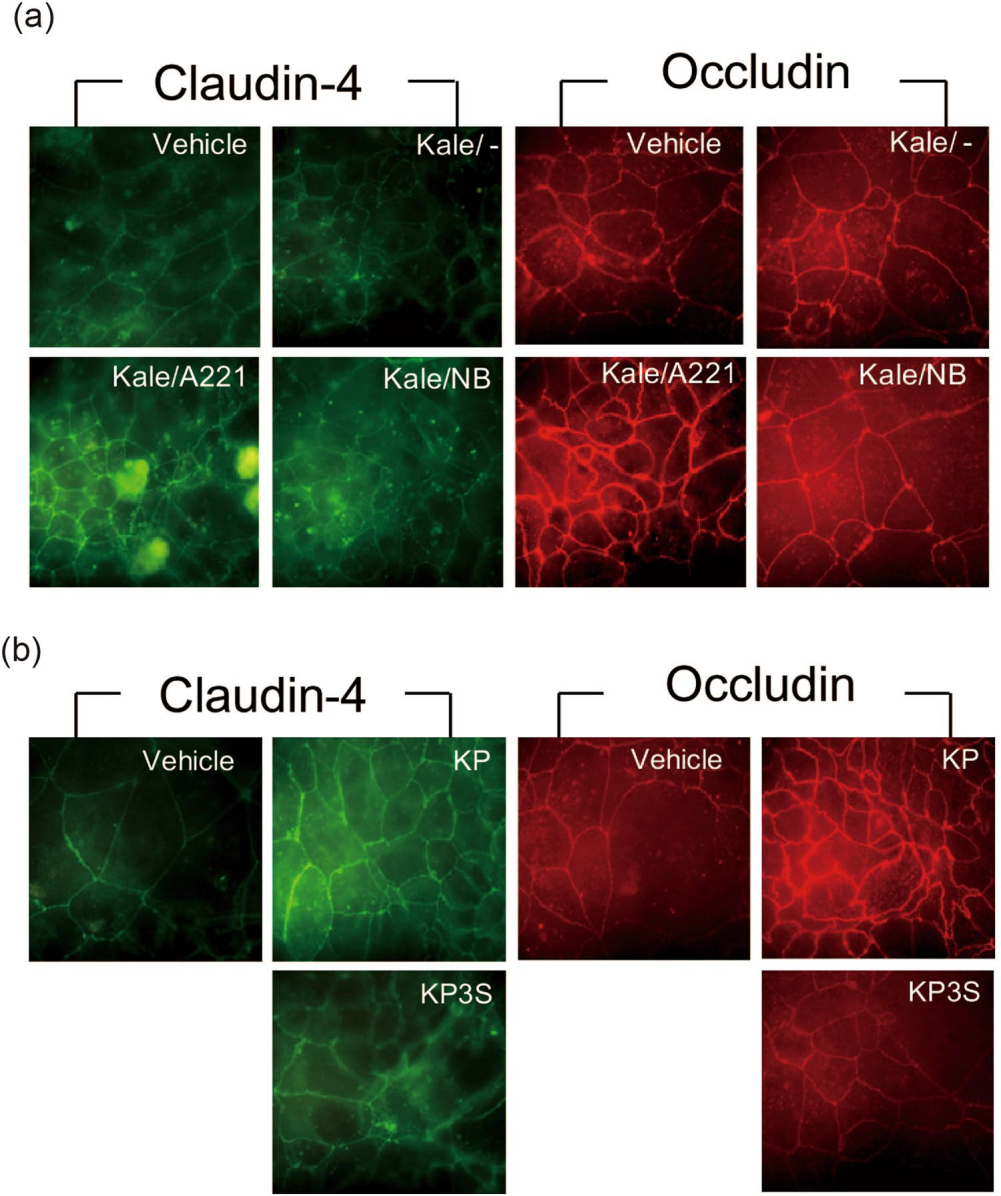
A221-treated kale water extract as well as KP promoted tight junction formation in a Caco-2 intestinal barrier model. (**a**) Differentiated Caco-2 cells were treated with each of the test samples for 6 h, followed by fixation and immunohistochemical staining using anti-claudin-4 and anti-occludin antibody as described in the Methods section. (**b**) In order to assess the important role of aglycone, either KP or KP3S was administered to Caco-2 cells and processed in the same manner as (**a**).

Because the involvement of unknown components from the A221 cell body was speculated, 50% ethanol extract of A221 was prepared and subjected to the same assay. However, there were no apparent changes in the TEER values with the treatment of A221 extract (data not shown), suggesting that the component of the A221 cell body does not contribute to the change in the TEER. Since KP in the A221-treated kale extract might be involved in the increase in the TEER value, Caco-2 cells were tested with either KP or KP3S, which is used hereafter as a model glucoside in kale, to determine whether or not aglycone was superior to its sophorosides in terms of increasing the barrier integrity. Figure 3b shows that KP significantly increased the TEER value in the Caco-2 model in a dose-dependent manner, whereas KP3S induced no apparent changes. In accordance with the above experiment, treatment with A221-treated kale extract but not with NBRC15889-treated kale extract nor with vehicle-treated kale extract clearly induced tight junction formation, as assessed by an immunohistochemical study using anti-claudin-4 and anti-occludin antibodies (Fig. 4a).

In the experiment using KP and KP3S, KP showed the clear accumulation of claudin-4 and occludin at the site of the tight junction, while KP3S did not show such changes (Fig. 4b). These results suggest that the hydrolysis of the disaccharide part in KP3S is needed to result in tight junction formation and barrier improvement *in vitro*, and the A221 strain is involved in such processes.

### Effects of A221 on the bioavailability of KP3S

**Figure 5:**
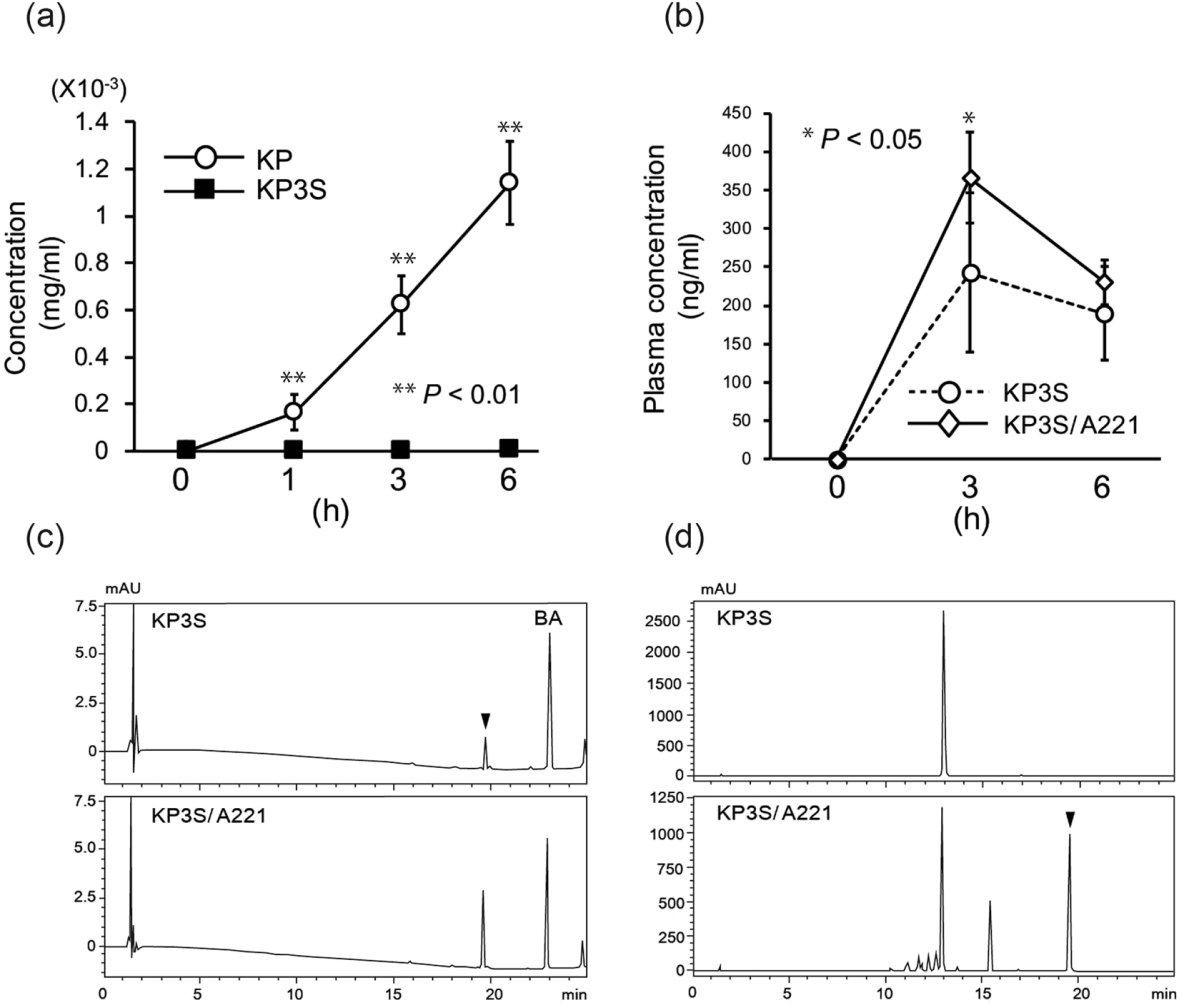
The effect of the A221 strain on the bioavailability of KP3S. (**a**) An *in vitro* study on the permeability of KP and KP3S using the Caco-2 intestinal barrier model was carried out. KP and KP3S were administered to Caco-2 cells on the apical side at 28.6 and 61 mg/ml, respectively, and the concentration of each chemical in the basolateral compartment was quantified by an HPLC analysis. **P < 0.01 vs. initial (0 h). (**b**) The effects of A221 administration on the bioavailability of KP3S were assessed using C57/B6 mice with the protocol described in the Methods section. Deconjugated KP in the plasma sample was quantified by an HPLC analysis at different time points. *P < 0.05 vs. KP3S. (**c**) Representative HPLC chromatograms of a plasma sample are shown for the group treated with only KP3S and the group treated together with A221. Arrowhead indicates the peak of deconjugated KP, and BA indicates the peak of Biochanin A. (**d**) The bioconversion of KP3S into KP was confirmed in microtubes by incubating A221 with KP3S in the same sample preparation for the *in vivo* administration experiment. Arrowhead indicates the peak of KP.

In addition to their influence on biological activities, the hydrolysis of glucosides has been considered a factor affecting the bioavailability of components like ginsenoside in ginseng^4^. Because A221 was found to convert KP3S into KP, the administration of the A221 strain might also modulate the bioavailability of KP3S. To investigate this possibility, the permeability of KP and KP3S was evaluated first using a Caco-2 model. As shown in Fig. 5a, the concentration of KP in the basolateral compartment increased in a time-dependent manner when the intestinal model was treated by KP at the apical side. In contrast, neither KP3S nor KP was detected in the basolateral compartment in the KP3S-treated group, suggesting that the sophoroside moiety has a hindering effect on the passive diffusion of KP3S through the Caco-2 cell layer.

Next, the direct effect of A221 strain on the bioavailability of KP3S was tested using male C57/B6 mice. After a 16-h fasting period, KP3S was orally administered to each mouse weighing approximately 20 g by gavage at 0.42 mg dissolved in 200 μl of water, which corresponds to the administration of 21 mg/kg KP3S. In order to assess the effect of the A221 strain, 25 mg of freeze-dried powder was administrated along with KP3S, and the whole plasma was collected at 0, 3 and 6 h for each experimental group. The plasma samples were then subjected to beta-glucuronidase and sulfatase treatment in order to liberate free aglycone, with the resulting KP quantified by an HPLC analysis. As shown in Fig. 5b and c, the plasma samples of the group that received the co-administration of KP3S with the A221 strain showed a significant increase in the amount of deconjugated KP at 3 h compared with the group treated without the A221 strain.

Based on the results of the *in vitro* permeability assay using Caco-2 cells mentioned above, the promotion of KP3S bioconversion by the A221 strain was strongly suspected to be involved in the improvement of its bioavailability. To confirm the reduction of KP3S and the appearance of KP, 0.42 mg of KP3S was co-incubated with 25 mg of A221 freeze-dried powder in microtubes for 3 h at 37 °C and then subjected to an HPLC analysis. Figure 5d shows the reduction of KP3S at the retention time of 12.5 min and the clear appearance of KP at 19.5 min by the treatment with A221 dry powder. These results indicate that A221 contributes to the bioavailability of KP3S by directly converting the substrate into the more permeable aglycone KP.

### Effects of long-term treatment of KP in a dermatological aging mouse model

**Figure 6:**
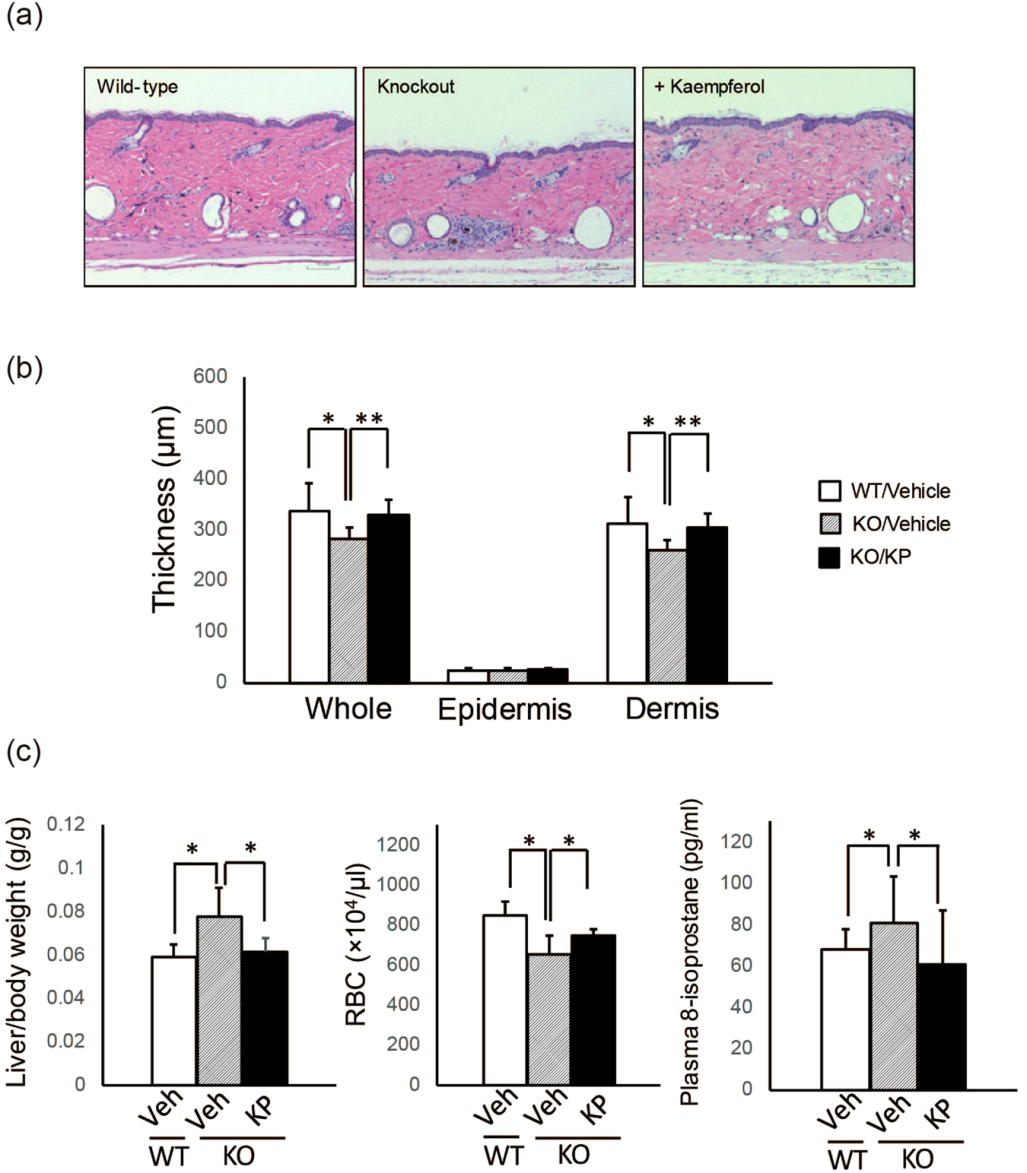
KP ameliorates the pathological phenotypes of *Sod1*^−/−^ mice. (**a**) KP was orally administered for eight weeks to male *Sod1*^−/−^ mice at 10 mg/kg, and skin sections were prepared. Hematoxylin and eosin staining was carried out, and representative images were shown for the different subjects in each groups. (**b**) The thickness of whole skin, epidermis, and dermis were quantified and compared among groups. (**c**) The effects of KP administration on the ratio of liver/body weight, the number of red blood cells, and the amount of 8-isoprostans were quantified and compared among groups. *P < 0.05 vs. KO Vehicle. **P < 0.01 vs. KO Vehicle.

To address the significance of KP in terms of the biological activity *in vivo*, the long-term administration of KP was carried out in hairless *Sod1*^−/−^ mice, a good mouse model of chronological aging including dermatological thinning^19–22^. Fatty liver is also reported in *Sod1-*deficient mice^23^. The oral administration of KP to these mice at 10 mg/kg for 8 weeks significantly improved the thickness of the overall skin, as shown in Fig. 6a,b. The thinning of the dermis was clearly attenuated by the treatment of KP. Furthermore, the liver weight, the number of red blood cells, and the levels of plasma 8-isoprostan, which is a marker of oxidative stress, were also significantly ameliorated compared with the group without KP treatment (Fig. 6c), indicating that KP has great potential as a biological active component for suppressing various age-associated pathologies caused by oxidative damage.

## Discussion

This study was designed to clarify the effect of the probiotic *Lactobacillus paracasei* A221strain on the properties of flavonoid glucosides, such as kaempferol-glucoside, from the perspective of functional improvement and the bioavailability of food-derived phytochemicals. In this study, this strain was found to convert KP3S into KP, and this process was suggested to be involved in the improvement of kale’s functionality in terms of intestinal barrier strengthening *in vitro*, since KP clearly increased the TEER value and tight junction formation whereas KP3S did not. This is consistent with the findings reported by Suzuki *et al*., who found that KP enhances the intestinal barrier function *in vitro*^24^. In view of the structural difference, KP possesses the free hydroxyl functional group at position 3 in the C-ring, while that of KP3S is used for the glucoside linkage. Additional studies will help clarify whether or not the hydroxyl functional group on the C-ring is participating in the barrier-enhancement of Caco-2 cell monolayer by KP.

Because approximately 10–15 μM of KP was contained in the A221-treated kale extract sample, as determined in an HPLC analysis, and the extent of increase in TEER value treated with 10 μM KP was still lower than that in the A221-treated kale group, other unknown components might also be involved in the functional improvement of kale water extract. As such, the cell body extract of A221 was also tested for the TEER analysis. However, no clear changes were observed. Further analyses are required to clarify other factors affecting the intestinal TEER value and tight junction formation induced by A221-treated kale water extract.

The ability of A221 to influence the bioavailability of KP3S was also investigated by an experiment using C57/B6 mice. The plasma samples were compared in order to measure the deconjugated KP between the experimental group that received the co-administration of A221 and KP3S and that without A221. The results revealed a significant increase in the amount of KP in the former group at 3 h post-administration. In addition, the permeability of KP was found to be superior to that of KP3S in Caco-2 barrier models. As reported by Makino *et al*., quercetin glucosides with unique disaccharides, such as gentiobioside, showed difficulty in being hydrolyzed by rat intestinal epithelium, suggesting that its intestinal uptake is relatively low^25^. Since KP3S possesses sophoroside, which has a unique beta-1,2 glycoside bond, KP3S may need to be hydrolyzed by intestinal bacterium before absorption by the intestine, like ginsenoside Rb1, which also possess the same sophoroside moiety at position 3 in its steroidal structure.

Substantial effort has been undertaken to improve the flavonoid bioavailability, and structural changes in flavonoid glucosides have been proposed as important factors influencing their absorption^26^. These findings suggest that the A221 strain may contribute to the bioavailability of KP3S by promoting the hydrolysis of flavonoid glucosides to achieve a more-permeable aglycone form.

Of note: the mice without A221 administration still showed deconjugated KP in their plasma samples (Fig. 5c). Given the diversity of commensal bacteria species in the intestine, certain types of intestinal bacteria might be responsible for the basal absorption of KP through the spontaneous hydrolysis of KP3S. Indeed, the fecal sample of mice without A221 treatment still showed KP3S-hydrolyzing ability (data not shown). However, a more detailed study will be required to confirm which bacteria have KP3S-hydrolyzing potential and to see if there are inter-individual differences in the bioavailability of KP3S, like ginsenoside saponin, of which the bioavailability depends on the individual intestinal environment^11,12^.

Finally, the biological effect of KP aglycone was investigated using *Sod1*^−/−^ mice through oral gavage administration, and clear ameliorations not only in the skin thinning but also in the liver weight as well as the number of red blood cells were observed. Although the precise mode of action by KP was not clear, these results suggest that promoting the production of KP from KP3S might have an important role in the enhancement of KP3S anti-aging activity *in vivo*.

The experimental results obtained in this study suggest that the function and bioavailability of flavonoid glucosides might be modulated by the intestinal microflora, like Rb1 ginsenoside saponin. It is documented the disaccharide part of rutin, a flavonoid glucoside with the rutinose part added at position 3 of quercetin, affects its bioavailability^27^. On one hand, it is reported that some *Lactobacillus* strains can hydrolyze the rutinose moiety of rutin, suggesting that such intestinal bacteria can modulate its bioavailability and pharmacological activities^28^. There is also a report by Uskova *et al*. that the biological activity of rutin was improved by co-administration with *Lactobacillus casei* 114001^29^. Furthermore, Hesperidin, a flavonoid glucoside contained in citrus fruits, possesses the same disaccharide as rutin at position 7 of hesperetin, and some of the *Bifidobacterium* strains are reported to show hydrolyzing activities for the rutinose moiety^30^. Thus, the roles of intestinal bacteria are diverse and considered important not only in the field of herbal medicine but also in the field of more general foods, including *Brassica* vegetables, as discussed in this paper.

Given these findings, this study provides for the first time insight into the potential benefit of certain *Lactobacillus* strains, particularly A221, in the improvement of the functionality and bioavailability of kaempferol-glucoside through the bioconversion into KP. Further analyses are desired to address whether or not KP is a central player in the biological activity of KP3S and whether or not the simultaneous administration with A221 strain generates beneficial outcomes in aging mouse models.

## Methods

### Chemicals

Standard chemicals of kaempferol (KP) and kaempferol-3-o-sophoroside (KP3S) were purchased from Extrasynthese (Lyon, France) and Chengdu Biopurify Phytochemicals, Ltd. (Sichuan, China), respectively. Biochanin A, beta-glucuronidase and sulfatase were all obtained from Sigma-Aldrich (St. Louis, MO, USA). Anti-claudin-4 (ab15104) and secondary antibodies of goat anti-rabbit IgG FITC (ab6717) were purchased from Abcam (Cambridge, UK). Anti-occludin antibody (#711500) and goat anti rabbit IgG Alexa fluor 594 (#R37117) were purchased from Thermo Fisher Scientific (Waltham, MA, USA).

### Microorganism and culture medium

*Lactobacillus paracasei* A221 was isolated from a fermented food as previously described^13^ and was provided by Nagase BeautyCare & Co., Ltd. This strain is deposited in the International Patent Organism Depository, National Institute of Technology and Evaluation (Kisarazu, Chiba 292-0818, Japan) as FERM BP-10123. The strain was cultured in Lactobacilli Inoculum Broth (Nissui, Tokyo, Japan) and seeded onto an MRS agar plate for colony isolation. *Lactobacillus paracasei* NBRC15889 was obtained from NBRC bank and propagated as the A221 strain. For the preparation of the freeze-dried powder of A221, cells were cultured for 48 h and collected by centrifugation, and the resulting cell pellet was then subjected to the freeze dry apparatus.

### Preparation of lactic acid bacteria-treated kale extracts

Raw kale leaf was crushed with a blender, and 120 g of blended leaf was soaked into 400 ml of distilled water. The mixture was heated at 80 °C for 1.5 h to extract water soluble components. The solution was then divided into 3 flasks, each of which contains 100 ml of kale water extract. For the preparation of A221-treated kale extract, 1 × 10^11^ cfu of the A221 strain was inoculated into the kale water extract and kept at 37 °C for 72 h. The mixture was freeze-dried once and then re-extracted with 50% ethanol. After evaporating the solvent, the residue was reconstituted with distilled water and freeze-dried to obtain powder of A221-treated kale extract. The same procedure was applied for the NBRC15889 strain-fermented kale extracts. For the non-treated kale extracts, autoclaved distilled water was added instead of lactic acid bacteria culture solutions. At the point of evaluation, all samples were reconstituted in 50% ethanol at 50 mg/ml for the stock solution.

### High-performance liquid chromatography (HPLC) analyses

To monitor the effect of lactic acid bacteria treatment on the component of the kale, the stock solution of each obtained extracts was subjected to an HPLC analysis (LCsolution; Shimazu, Kyoto, Japan) using a 3.9 × 150-mm μBondasphere C18 column (Waters, Milford, MA, USA). The following gradient elution was used: solvent A, 2.0% acetic acid; solvent B, acetonitrile. The following gradient system was used: from 5% B at 0 min to 20% B at 10 min and then to 80% B at 25 min. HPLC was performed at a flow rate of 1.0 ml/min and monitored at 365 nm. On the HPLC analysis, the peak of KP was confirmed at 19.5 min. To identify the peak at 8.2 min, the water extract of kale leaf was subjected to polyamide C-200 (Wako Pure Chemical Industries, Osaka, Japan) column chromatography for purification. The fraction containing the peak was then separated by column chromatography using TOYOPEARL HW40S (Tosoh Bioscience, Tokyo, Japan). LC-MS^3^ was conducted for the purified compound, and a parent ion of m/z 771 as well as fragmented peak ions of m/z 609, 429 and 285 indicating KP3S7G were all measured in negative-ion mode according to the report by Ferreres *et al*.^16^.

To investigate the effect of lactic acid bacteria on the metabolism of KP3S, lactic acid bacteria strains were inoculated in 4 ml of Lactobacilli Inoculum Broth (Nissui) and cultured for 72 h. At the end of the culture, 1 ml of bacteria suspension was transferred to a 1.5-ml tube and centrifuged to obtain a cell pellet. The standard KP3S was reconstituted in autoclaved distilled water at 50 µg/ml, and 100 µl was added directly to the cell pellet. The samples were kept at 37 °C for 72 h, adjusted to 50% ethanol concentration, and finally subjected to the HPLC analysis.

Freeze-dried powder of A221 was also prepared in the metabolism analysis of KP3S in conjunction with the experiment on the bioavailability of KP3S *in vivo*. In this experiment, 0.42 mg of KP3S was treated with or without 25 mg of A221 freeze-dried powder in 200 µl of autoclaved water and incubated for 3 h at 37 °C in 1.5-ml microtubes. After that, the samples were adjusted to 50% ethanol concentration and subjected to an HPLC analysis.

### The evaluation of the intestinal barrier function *in vitro*

Caco-2 cells obtained from ATCC (Manassas, VA, USA), were grown in Dulbecco’s modified Eagle’s medium (GIBCO; Thermo Fisher Scientific) supplemented with 10% fetal bovine serum (FBS), 50 μg/ml of streptomycin and 50 μg/ml of penicillin at 37 °C under 5% CO_2_. For the intestinal barrier evaluation, Caco-2 cells were seeded onto a 12-well Thincerts plate (Greiner Bio-one, Monroe, NC, USA) at 1 × 10^5^ cells/well. Cells were grown for 14 days for differentiation, with the culture medium replaced every 3 days. Once the *in vitro* intestinal barrier model had been established, the culture medium containing either non-treated kale extract, A221-treated kale extract or NBRC15889-treated kale extract was added to the cup of the cell monolayer and incubated for the indicated period. For the vehicle group, ethanol was added at the final concentration of 1%. The TEER values were measured using Millicell (Millipore, Billerica, MA, USA) at each interval, and the percentage of increase compared to the initial values was recorded. For the evaluation of KP and KP3S, the same procedure was applied.

For the assessment of the permeability using the Caco-2 barrier model, KP and KP3S were reconstituted in DMEM at 28.6 and 61 mg/ml, respectively, both of which were adjusted to 100 μM. Each sample (500 µL) was then applied to the apical side of the Thincert wells. Basolateral samples (100 µL) were then collected at the indicated time intervals, and ethanol was added to each sample to make a 50% ethanol concentration. The samples were then subjected to an HPLC analysis to quantify the amount of KP or KP3S in the basolateral culture medium.

### Immunohistochemistry

Caco-2 cells were seeded into 2-well slide chambers (NUNC) at 1 × 10^5^ cells/well and subjected to differentiation. After differentiation, the cells were exposed to either non-treated kale extract, A221-treated kale extract or NBRC15889-treated kale extract at 1 mg/ml for an additional 6 h. Ethanol was used at the final concentration of 1% for the vehicle control group. KP and KP3S were added to the cells at 100 μM and DMSO was used at the final concentration of 0.1% for the vehicle control group. The cells were then fixed with an ice-cold mixture of acetone and methanol (1:1). After wash out with PBS, the cells were treated with PBS containing 5% goat serum for 2 h at room temperature to block nonspecific signals. Rabbit anti-claudin-4 or rabbit anti-occludin was added to the cells at 1:200 overnight, after which secondary anti-body treatment (goat anti-rabbit IgG FITC for claudin-4 and goat anti rabbit IgG Alexa fluor 594 for occludin) was performed for 2 h at room temperature. Once slides were prepared, the cells were imaged at 300× by fluorescent microscopy.

### *In vivo* experiment for the bioavailability of KP3S

All of the animal experiments performed in this study were approved by the ethics committee of Chiba University based on their internal guidelines. After a 1-week acclimatization period, all male C57/B6 mice (C57BL/6NJcl; CLEA Japan, Tokyo, Japan) were subjected to a 16-h fasting period. After that, they were subdivided into two groups. One group received 0.42 mg of KP3S, which was reconstituted in 200 μl of autoclaved distilled water, by a single gavage treatment. The other group received KP3S together with 25 mg of A221 freeze-dried powder, which was also reconstituted in water with KP3S. Whole blood samples were then collected at 0, 3 and 6 h post-administration, followed by plasma preparation (n = 6 per each time points). Each plasma sample was adjusted to 400 μl with distilled water and spiked with Biochanin A as an internal standard at a final concentration of 0.02 mg/ml^31^. After adding 0.1 volume of 0.58 M acetic acid, beta-glucuronidase and sulfatase were added to each plasma sample at final concentrations of 4 × 10^6^ units/L and 1.5 × 10^5^ units/L, respectively, and incubated at 37 °C for 2 h for deconjugation.

Each sample was then subjected to solvent extraction with 7.5 volume of acetone and dried under N_2_ gas. Finally, the extracts were reconstituted with 400 μl of ethanol and subjected to an HPLC analysis. For the standard, sequential dilutions of KP were prepared in 400 μl of distilled water, and Biochanin A was spiked into each dilution. The same procedure as for plasma was applied to the standard preparation, which was then subjected to an HPLC analysis. Deconjugated KP was quantified, and the concentration in the plasma was calculated after adjustment, with the dilution rate depending on the initial plasma sample volumes.

### The evaluation of the bioactivity of KP in hairless *Sod1*-deficient mice

The method for preparing hairless *Sod1*^−/−^ mice has been described elsewhere^19^. For the experiment of KP *in vivo*, five-week-old male mice were divided into three groups (n = 8 mice per group): a wild-type vehicle control group, *Sod1*^−/−^ vehicle control group and *Sod1*^−/−^ KP group. KP was administered orally with gastric gavage at 10 mg/kg daily for 8 weeks. Distilled water was administered to mice in vehicle control group. At the end of the administration, mice were sacrificed, and whole blood was collected. The number of red blood cells was then measured with an automatic hematology analyzer (Celltac MEK-4150; Nihon Kohden, Tokyo, Japan). In addition, the liver weight was measured to evaluate the effect of KP. Skin specimens was subjected to hematoxylin and eosin staining to measure the thickness of the epidermis and dermis.

### Statistical analyses

For the *in vitro* intestinal barrier experiment, Dunnett’s test was conducted between vehicle control and tested groups at each time interval. For the experiments of *in vivo* bioavailability of KP3S, Student’s *t*-test was applied to evaluate the difference between administration with KP3S and that with the A221 strain. In the experiment using hairless *Sod1*^−/−^ mice, statistical analyses were performed by a one-way analysis of variance followed by Tukey’s test. *P* < 0.05 was considered significant.
